# PROTOCOL: Methods used in the development, production and updating of evidence and gap maps: A scoping review

**DOI:** 10.1002/cl2.1402

**Published:** 2024-04-28

**Authors:** Fiona Campbell, Ruth Wong, Jennifer L. Llewellyen, Akvile Stoniute, Fiona Pearson, Melissa Bond

**Affiliations:** ^1^ Population Health Sciences Institute Newcastle University Newcastle UK; ^2^ School for Health and Related Research University of Sheffield Sheffield UK; ^3^ EPPI Centre University College London London UK

**Keywords:** evidence and gap maps, evidence synthesis, mapping reviews, scoping review, systematic review

## Abstract

Evidence and gap maps (EGMs) are an increasinly popular approach used in evidence synthesis. As an approach they address broad research questions, describing the existing evidence base, highlighting evidence gaps and providing an interactive visual tool for knowledge users. The purpose of this methodological study is to explore the the processes used in the development of EGM's and how they are reported. The aim is to better understand current practice and identify where clearer guidance is needed to support their production.

## 1 BACKGROUND

Evidence and Gap Maps (EGMs) sit within a family of evidence synthesis methods that seek to address broader research questions, such as; what interventions have been evaluated in the treatment of COVID‐19 (Campbell et al., 2023), and what review level evidence has been published exploring elder abuse (Mikton et al., 2022). These types of broad research questions contrast with the focused questions in an effectiveness systematic review where a PICO framework (population, intervention, comparator, and outcomes) helps to clearly specify the specific intervention that is being evaluated, in which population, against which comparator and for what outcomes. Broader review questions may use an alternative question framework that allow for concepts or multiple interventions or multiple study designs to be considered in one review. These types of evidence synthesis approaches have gained greater prominence and have a particular value in supporting decision making where a wider understanding of an area is needed and gaps in knowledge need to be readily identified in order to direct future research priorities (Snilstveit et al., 2013). Within the context of the COVID‐19 pandemic, where rapid evidence was needed and new research was being conducted and published within short time frames, evidence synthesis approaches that allowed the range of evidence becoming available and freely accessible to a wide audience were needed.

EGMs are a valuable tool in meeting these needs. They do not synthesise existing evidence, but by locating, categorising, coding and presenting the evidence in an interactive web‐based tool (White et al., 2020), with links to the primary research, they offer a valuable visualisation of existing evidence.

A unique feature of these approaches is that the matrix into which the evidence is plotted, is generated a priori. Consultation with stakeholders and the use of theoretical frameworks will inform the construction of the dimensions within the framework. For example, when wanting to locate evidence for interventions to reduce elder abuse, all potential interventions are identified in the design of the matrix. The map can therefore show, not only the available evidence, but also the gaps where evidence is lacking.

A barrier to the use of evidence in decision making is that systematic reviews are out of date or fail to be relevant to a particular context where a decision is being made. Advances in technologies and processes have created the foundations for frequent or ‘living’ approaches to updating. During the COVID‐19 pandemic, clinical practice and policy decisions needed to be made quickly and be informed by the explosion in COVID‐19 research. Several groups used the foundational work on living approaches in systematic reviews to launch COVID‐19 living systematic reviews, guidelines and EGMs (e.g., Lorenc et al., 2023). For example, both the WHO and NICE, transitioned their COVID‐19 guidelines into living modes.

Both living approaches and EGMS are still new and evolving approaches. There is little in the way of guidance and methods of creating a living map and approaches to updating maps. Although COVID‐19 was a catalyst to progressing some of these methods, the frequency and implications of updates on policy and practice may vary from topic to topic. Recommendations for methods are likely to therefore need tailoring to the review topic (Elliott et al., 2014; Elliott et al., 2021).

An EGM, with the use of filters to focus a search of existing evidence can therefore be particularly valuable. For example, the Youth Endowment Foundation EGM of interventions to prevention young people getting involved in violence (https://youthendowmentfund.org.uk/wp-content/uploads/2022/04/Map-21-Apr-22-2.html) allows users to locate evidence conducted in particular settings. Living reviews have evolved as ways to address the problem of evidence becoming out of date and redundant. EGMs, as they do not synthesise the included studies, are one tool where the development of a living evidence product faces fewer practical hurdles. The fact that these are also web‐based is a feature that means regular updates are practically easier.

This review seeks to explore what methods of developing, maintaining and updating EGMs are currently used, and how.

## 2 REVIEW QUESTIONS

(1) What methods are currently used in the development, production and updating of EGMs?
(2) To what extent do EGMs adhere to recommended guidance (Campbell, PRISMA‐ScR)?
(3) What procedures are in place for updating and maintaining EGMs?
(4) What methodological guidance are being cited to support the approaches used in the EGM process?

## 3 METHODS

We will use a scoping review methodology to address our broad research question (Campbell et al., 2023). It will draw on methods described by James et al. (2016), Arksey and O'Malley (2005) and Levac et al. (2010); including the location, screening, data extraction, coding and description of findings. The review will comply with PRISMA‐ScR (Tricco et al., 2018) reporting guidance.

### 3.1 Locating the EGMs

Interactive visual representation of evidence gap maps can be created by platform providers such as EPPI Mapper (Digital Solution Foundry and EPPI‐Centre, 2022) and can be displayed on a public‐facing website. It is unclear whether all maps will have a related journal publication describing the map and to our knowledge, there is no search filter on how to find evidence gap maps online. EGMs created using EPPI Reviewer and EPPI Mapper will be systematically located using the Google search engine. In addition, through collaboration with the EPPI Centre, we will undertake web‐searching for public‐facing maps that have been created using the EPPI Mapper tool. We will identify the number of unique maps (some maps have several URLs) and search to obtain the supporting documentation describing the map such as associated publication, published report, technical guide and web resource. Other maps may be identified by reference tracking of the supporting documentation of included maps.

### 3.2 Inclusion and exclusion criteria

#### 3.2.1 Inclusion criteria

- EGMs created by EPPI Mapper (Digital Solution Foundry and EPPI‐Centre, 2022)
- Public‐facing online maps (with a URL)
- Published in English

#### 3.2.2 Exclusion criteria

- EGMs created using other platforms such as the 3ie tool or EviMap.

### 3.3 Data extraction

Data concerning the presentation of the map, reporting of methods and methods for updating the map will be gathered by two reviewers working independently. Differences between the reviewer coding will be explored and discussed. Where necessary, a third reviewer will be included in order to achieve consensus. In particular, the following information will be extracted, where available:

General

(1) Website and host site for the map
(2) Authors
(3) Date of launch and map number (if applicable)
(4) Who the intended end‐users are i.e. decision makers and/or researchers
(5) Location of accompanying protocol and report (links from map)
(6) Team size, skills and roles
(7) Software tools used

Review question and purpose

(8) Research question/topic addressed by the EGM
(9) Type of evidence included in the EGM
(10) Date coverage of the evidence in the map e.g. from 2010 to present
(11) Description of methods used
  (a) Description of search strategy
  (b) Stakeholder engagement
  (c) Number of databases searched
  (d) Inclusion criteria defined
  (e) Types of studies included in the map
  (f) Methods of screening and data coding
  (g) Quality appraisal
  (h) Methods cited
(12) Limitations of the evidence in the map e.g. language bias, exclusion of unpublished literature reported

Updating and/or living maps

(13) Any indication if/when the map will be updated
(14) Description of ‘living’
(15) Methods for updating
(16) Use of automation in updating
  (a) Searching
  (b) Screening
  (c) Coding
  (d) Quality assessment
(17) Funding for updating
(18) Team roles in updating
(19) Transparency of reporting when last search was undertaken

An area that will not be addressed in this mapping review, but nevertheless considered by the authors, are considerations of equity, and how socially stratifying factors are accounted for in the map. This question is being addressed in a concurrent scoping review (Khalil et al., 2024).

### 3.4 Synthesis of findings

The data extracted will be collated, tabulated and described narratively.

The framework for the description of the methods used will follow the research questions and objectives of this review.

- What methods are currently used in the development, production and updating of EGMs?
- To what extent to EGMs adhere to recommended guidance (White et al., 2020; PRISMA‐ScR)?
- What procedures are in place for updating and maintaining EGMs?
- What methods are being cited by EGM authors guiding the research process?

## 4 CONCLUSION

This scoping review will endeavour to describe the current state of practice in the development of evidence gap maps, a burgeoning approach to evidence synthesis and one that has particular value in policy making (Snilstveit et al., 2013). It is an approach that is being widely adopted by organisations that fund research and seek to inform policy (e.g., 3ie, Youth Endowment Fund, Unicef). We want to explore the extent to which these outputs are supported by published protocols, transparent and rigorous methodology, and how efforts to maintain their relevance are ensured. The emergence of ‘living maps’ will be included in our analysis and we will describe how ‘living’ is defined and operationalised in these reviews. With their increasing popularity and their importance in shaping policy, ensuring these outputs are reliable and trustworthy sources of evidence is important. A good understanding of current practice will provide a sound basis for further development of existing reporting standards such as PRISMA‐ScR which currently do not address the development of EGMs.

We acknowledge that limiting our inclusion criteria to those EGMs produced using EPPI Mapper is a limitation of this review. Other mapping tools exist and are used within environmental science (https://www.oxsrev.org/systematic-mapping) and international development (https://www.3ieimpact.org/). However, the tools to support the development of these EGMs are created by specific review groups with a particular topic focus, which limits their adaptability to other topic areas, and they are not publicly available. EGMs created by EPPI Mapper can be adapted to any topic area and hosted on any website.

## CONTRIBUTIONS OF AUTHORS

FC, FP and MB both have expertise in producing evidence and gap maps and in using EPPI Reviewer and EPPI Mapper. FC conceived the project, FC, RW and MB prepared the protocol. FC, AS and JL will undertake the data extraction. RW has expertise as an Information Specialist and will design the search strategies. All authors reviewed and agreed the protocol.

## DECLARATIONS OF INTEREST

FC, RW, JL, AS and FP have no conflicts of interests to declare. MB works at the EPPI Centre that created the EPPI Reviewer software but does not receive any benefits personally from it, as UCL is a non‐profit organisation.

## PRELIMINARY TIMEFRAME

The review will be submitted by September 2024.

## PLANS FOR UPDATING REVIEW

FC will be responsible for updating the review. Funding to support updates beyond 2025 will be sought.

## SOURCES OF SUPPORT

### Internal sources

None.

### External sources

This work was supported by the UK Prevention Research Partnership (MR/S037578/2, Meier).
